# Fibrinogen to Albumin Ratio as Early Serum Biomarker for Prediction of Intra-Hospital Mortality in Neurosurgical Intensive Care Unit Patients with Spontaneous Intracerebral Hemorrhage

**DOI:** 10.3390/jcm11144214

**Published:** 2022-07-20

**Authors:** Michael Bender, Kristin Haferkorn, Shahin Tajmiri-Gondai, Eberhard Uhl, Marco Stein

**Affiliations:** Department of Neurosurgery, Justus-Liebig-University Gießen, Klinikstraße 33, 35392 Gießen, Germany; kristin.haferkorn@neuro.med.uni.giessen.de (K.H.); shahin.tajmiri-gondai@neuro.med.uni-giessen.de (S.T.-G.); eberhard.uhl@neuro.med.uni-giessen.de (E.U.); marco.stein@neuro.med.uni-giessen.de (M.S.)

**Keywords:** fibrinogen to albumin ratio, serum biomarker, intensive care unit treatment, intracerebral hemorrhage, intrahospital mortality

## Abstract

Background: The prognostic value of the fibrinogen to albumin ratio on intrahospital mortality has been investigated in patients with cardiovascular disease, cancer, sepsis, and ischemic stroke; however, it has not been investigated for neurosurgical patients with spontaneous intracerebral hemorrhage (ICH). The present study investigates the impact of the fibrinogen to albumin ratio upon admission for intrahospital mortality in neurosurgical intensive care unit (ICU) patients with spontaneous ICH. Methods: A total of 198 patients with diagnosis of spontaneous ICH treated from 10/2008 to 12/2017 at our ICU were retrospectively analyzed. Blood samples were drawn upon admission, and the patients’ demographic, medical data, and cranial imaging were collected. Binary logistic regression analysis was performed to identify independent prognostic factors for intrahospital mortality. Results: The total rate of intrahospital mortality was 35.4% (*n* = 70). In the multivariate regression analysis, higher fibrinogen to albumin ratio (OR = 1.16, CI = 1.02–1.31, *p* = 0.03) upon admission was an independent predictor of intrahospital mortality in neurosurgical ICU patients with ICH. Moreover, a fibrinogen to albumin ratio cut-off level of >0.075 was related to increased intrahospital mortality (Youden’s index = 0.26, sensitivity = 0.51, specificity = 0.77). Conclusion: A fibrinogen to albumin ratio > 0.075 was significantly associated with increased intrahospital mortality in ICH patients.

## 1. Introduction

Despite advances in intensive care medicine and surgical treatment, spontaneous intracerebral hemorrhage (ICH) is still associated with high rates of morbidity and mortality [1,2,3,4,5,6,7]. The prediction of neurological outcome and mortality is an elementary part of daily business for intensive care unit (ICU) healthcare professionals. Therefore, the most commonly used predictors for poor outcome after ICH include lower level of consciousness, advanced age, lower initial Glasgow Coma Scale (GCS), larger peri-hemorrhagic edema, presence of hydrocephalus and intraventricular hemorrhage (IVH) and larger volume and expansion of ICH [8,9,10,11,12,13]. Additionally, to these established predictors, serum biomarkers could be helpful alternatives to predict intrahospital outcome and mortality. Several studies have indicated an association of elevated level of cortisol, troponin I, C-reactive protein (CRP), white blood cell count, blood glucose level, and CRP to albumin ratio with poor outcome after ICH [6,14,15,16,17].

Inflammation and acute stress are important elements of the pathophysiology pathway of acute ICH and secondary brain injury [17]. The fibrinogen to albumin ratio, as an inflammatory serum biomarker, has been identified in several studies as a predictor of poor outcome and adverse events in patients with cardiovascular diseases, cancer, sepsis, and stroke [18,19,20,21,22,23,24,25,26,27]. The acute-phase protein fibrinogen is an important part of the coagulation cascade as well as an indicator of systemic inflammation [20,26,27]. Castellanos et al. reported an association of good outcome and low fibrinogen level in non-surgically treated patients with medium to large spontaneous ICH [27]. In contrast, albumin is an anti-acute phase protein and an important clinical parameter for the current liver function as well as the nutrition status of patients. Lower levels of serum albumin upon admission are associated with increased mortality in ICH patients [6]. The impact of the fibrinogen to albumin ratio in patients with ICH admitted to the neurosurgical ICU is still unknown. However, the fibrinogen to albumin ratio, as an early serum biomarker, could be helpful for the early identification of ICH patients with a high risk of intrahospital mortality as well as useful to improve prognostication and further decisions with respect to ICU treatment. The present study was conducted to investigate the impact of the fibrinogen to albumin ratio on intrahospital mortality among neurosurgical ICU patients with spontaneous ICH.

## 2. Materials and Methods

### 2.1. Study Design and Population

In this retrospective study, all patients with spontaneous ICH who were admitted to the neurosurgical ICU of the Neurosurgical Department of the University Hospital Giessen between October 2008 and December 2017 were analyzed (*n* = 750). All patients who received inpatient treatment in the ICU for at least 24 h and whose fibrinogen and albumin level were determined upon admission were included (*n* = 198). The exclusion criteria were defined as follows: (A) ICH due to vascular malformation (*n* = 126), neoplasia (*n* = 86), or trauma (*n* = 117); (B) presence of chronic acute and/or acute liver failure (*n* = 23); (C) evident hyperfibrinolysis (*n* = 3); and (D) age under 18 years. The Ethics Committee of the Justus-Liebig-University, Giessen, Germany (No. 95/17) approved the study protocol.

### 2.2. Data Collection

The baseline data, including age, sex, body mass index, GCS, the National Institutes of Health Stroke Scale (NIHSS), acute physiology and chronic health evaluation II (APACHE II) score, and duration of hospital stay, as well as comorbidities, premedication, serum biomarkers, and computed tomography scan upon admission were evaluated [28,29,30]. In addition, cardiopulmonary parameters (CP) within the first 24 h, treatment regime, and intrahospital outcome mortality at discharge were collected from the patient’s electronic medical records. In all included patients, the presence of heart failure, chronic arterial hypertension, history of ICH or ischemic stroke, coronary artery diseases, cardiac arrhythmia, history of cardiac/cardiosurgical intervention, chronic obstructive pulmonary diseases, chronic renal insufficiency, cancer, and diabetes mellitus were assessed as comorbidities. Furthermore, analyzation of premedication was comprised of the absence of premedication, antihypertensive, antiobstructive, antidiabetic, and antiplatelet agents, new oral anticoagulants, and vitamin K antagonists.

### 2.3. Treatment

After the initial clinical examination in the emergency room, all patients were transferred to the computed tomography (CT) suite for confirmation of ICH diagnosis. According to their clinical and radiological conditions, the patients were treated at the neurosurgical ICU for at least 24 h, either immediately or after urgent surgery. A neurosurgeon consultant evaluated the indication for conservative or further surgical treatment (e.g., the evacuation of ICH, external ventricular drain (EVD), decompressive craniectomy with evacuation of ICH or decompressive craniectomy). All patients were analyzed regarding the treatment regime performed.

### 2.4. Intensive Care Unit Treatment

An arterial oxygen partial pressure above 100 mmHg and a systolic arterial blood pressure of 120–140 mmHg were defined as targets of CP during the first 14 days of ICU treatment. A 3-lead electrocardiogram (B. Braun, Melsungen, Germany), a pulse oximeter (Nellcor adult SpO_2_ sensor; Covidien LLC, Mansfield, MA, USA), and an invasive blood pressure measurement catheter (Combitrans Monitoring Set arteriell; B. Braun, Melsungen, Germany) were used for cardiopulmonary monitoring in all ICH patients. Furthermore, a central venous catheter (Arrow International, Inc., Reading, PA, USA) was placed in all ICH patients for the administration of intravenous drugs. Analysis of blood gas (ABL800 FLEX; Radiometer, Krefeld, Germany and Copenhagen, Denmark) was carried out every four hours by taking arterial blood samples. Additionally, in cases of respiratory insufficiency or a GCS score of less than 9, endotracheal intubation and mechanical ventilation were initiated (Servo-I; Maquet, Rastatt, Germany). Sufentanil (35–100 µg/h), in combination with propofol (200–500 mg/h) or midazolam (5–40 mg/h) was used for continuous analgosedation.

### 2.5. Cardiopulmonary Parameters

Analysis of CP included intubation status and body temperature on admission as well as systolic blood pressure, heart rate, positive end-expiratory pressure level (PEEP), arterial oxygen partial pressure, inspiratory oxygen fraction (FiO_2_) and mean norepinephrine application rate (NAR) during the first 24 h of the ICU treatment. All CP were stored in our digital ICU data-recording system in a period of 5 min.

### 2.6. Serum Biomarkers

After the patients’ admission to the neurosurgical ICU, a blood sample was drawn. Hemoglobin level in g/dl (XE 5000 Hematology Analyzer, Sysmex, Germany), white blood cell count in giga/L (XE 5000 Hematology Analyzer, Sysmex, Norderstedt, Germany), hematocrit level in % (XE 5000 Hematology Analyzer, Sysmex, Norderstedt, Germany), blood glucose level in mg/dl (ADVIA Chemistry XPT^®^, Siemens, Berlin, Germany), troponin I in µg/dL (ADVIA Centaur XPT^®^, Siemens, Berlin, Germany), cholinesterase in U/L (ADVIA Chemistry XPT^®^, Siemens, Berlin, Germany), serum lactate level in mmol/L (ADVIA Chemistry XPT^®^, Siemens, Berlin, Germany), C-reactive protein (CRP) in mg/L (ADVIA Chemistry XPT^®^, Siemens, Berlin, Germany), cortisol level in µg/dL (ADVIA Centaur XPT^®^, Siemens, Berlin, Germany), prothrombin time in % (Atellica^®^ COAG 360 System, Siemens, Berlin, Germany), albumin level in g/L (ADVIA Chemistry XPT^®^, Siemens, Berlin, Germany), thyroid stimulating hormone in mU/L (ADVIA Centaur XPT^®^, Siemens, Berlin, Germany), partial thromboplastin time in sec (Atellica^®^ COAG 360 System, Siemens, Berlin, Germany), antithrombin III in %/NORM (Atellica^®^ COAG 360 System, Siemens, Berlin, Germany), and fibrinogen in g/L (Atellica^®^ COAG 360 System, Siemens, Berlin, Germany) were evaluated as serum biomarkers on admission in all included patients. In addition, the CRP toalbumin ratio and fibrinogen to albumin ratio were calculated by the division of the initial CRP level by the albumin level and the initial fibrinogen level by the albumin level upon admission, respectively.

### 2.7. Computed Tomography Scan

The CT scans were evaluated by two independent investigators (i.e., S.T.-G. and M.B.). There was no significant difference concerning presence of hydrocephalus and IVH or volume of ICH volume between the investigators. The localization of the ICH was analyzed from the CT scan upon admission and stratified in deep and lobar supratentorial as well as infratentorial localization. The ICH volume was calculated by using the formula A × B × C/2. In addition, the evidence of an IVH (Graeb score > 1) and hydrocephalus (Evans’ Index > 0.3) was assessed and analyzed [31,32].

### 2.8. Intra-Hospital Outcome and Mortality

The intrahospital outcome and mortality were assessed by using the Modified Rankin Scale (mRS) at discharge [33]. The entire study population were stratified in survivor (mRS = 1–5) and non-survivor (mRS = 6)

### 2.9. Statistical Analysis

The entire cohort was stratified into nonsurvivors and survivors within inpatient treatment. Data are expressed as the mean ± standard deviation for the parameters with normal distributions and the median and interquartile range (IQR) for non-normal distributed parameters. The Student’s *t* test or Mann–Whitney U-Test were used for univariate analysis and the Chi-square test to evaluate differences in binary variables between survivors and nonsurvivors. A *p*-value of <0.05 was defined as the level of significance. All parameters that reached the level of significance in the univariate analysis were further investigated using a multivariate binary logistic analysis with a forward stepwise method. The Statistical Package for the Social Sciences (SPSS) version 15.0 for Windows (Version 15.0; SPSS Inc., Chicago, IL, USA) was used for data analysis. In addition, a cut-off level for the fibrinogen to albumin ratio level concerning intrahospital mortality was calculated. R statistical software (Version 3.4.1, RCore Team 2017, Dormagen, Germany) was used to evaluated the area under the curve and the Youden’s index in a receiver operating curve (ROC) analysis. Additionally, all independent predictors of intrahospital mortality were correlated with the fibrinogen to albumin ratio to identify significant correlations by using the Spearman correlation.

## 3. Results

### 3.1. Main Characteristics

A total of 198 patients with a mean age of 68.7 ± 13 years (age range: 33–93 years) were included in this study, of whom 87 (43.9%) were women. Upon admission, a median GCS score of 6.5 (IQR: 3–11), APACHE II score of 18 (IQR: 14–21), NIHSS score of 15 (9–25) and mRS score of 5 (3–5) were observed. Chronic arterial hypertension (60.1%) and cardiac arrhythmia (19.3%) were the most common comorbidities. A total of 102 patients (51.1%) were taking a pre-existing medication, most commonly antihypertensive drugs (48.5%) and vitamin K antagonists (21.2%), as shown in Table 1. The total study population required an average NAR of 0.03 ± 0.05 mg/kg/min and a FiO_2_ of 37.5 ± 13.5 to achieve the cardiopulmonary targets. In addition, in 141 patients (71.2%), intubation and mechanical ventilation were indicated within the first 24 h. The mean fibrinogen and albumin levels upon admission were 3.3 ± 1.1 g/L and 38 ± 5.8 g/L, respectively, with a calculated fibrinogen to albumin ratio of 0.9 ± 0.03. A medical treatment was carried out in 67 (33.8%) patients, and 131 patients (66.2%) required additional surgical treatment, in which evacuation of ICH (34.4%) occurred most frequently. Analyzation of computed tomography scan revealed a mean ICH volume of 61.2 ± 46.6 cm^3^ (range: 1.0–219.6 cm^3^), and 142 patients (71.7) presented an IVH. In addition, the most frequent localization of ICH was deep supratentorial, and 102 patients (51.5%) suffered from hydrocephalus.

### 3.2. Intrahospital Mortality and Outcome

The total study population had a median mRS of 5 (IQR: 4–6) at discharge and a mortality rate of 35.4%. Advanced age (*p* < 0.0001), male gender (*p* = 0.04), lower initial GCS score (*p* < 0.0001), higher APACHE II score (*p* < 0.0001), higher NIHSS score (<0.0001), shorter length of hospital stay (*p* < 0.0001), pre-existing medication (*p* = 0.007), consumption of antihypertensive medication (*p* = 0.02), and lower body temperature upon admission (*p* < 0.0001) was significantly associated with intrahospital mortality. The group of nonsurvivors exhibited initially lower cholinesterase (*p* = 0.008), albumin (*p* = 0.002), and prothrombin time (*p* = 0.003) levels, and a lower rate of infratentorial localization of ICH (*p* = 0.04), as well as a higher rate of conservative medical treatment (*p* = 0.003), larger ICH volume (*p* < 0.0001), higher incidence of IVH (*p* = 0.03), higher level of partial thromboplastin time (*p* = 0.01), higher CRP to albumin ratio (*p* = 0.02), and higher fibrinogen to albumin ratio (*p* = 0.002), as illustrated in Table 2.

In a multivariate binary analysis, advanced age (OR = 1.07, 95% CI: 1.03–1.1, *p* < 0.0001), lower GCS (OR = 0.75, CI = 0.67–0.85, *p* < 0.0001), higher APACHE II score (OR = 0.71, 95% CI = 0.63–0.81, *p* < 0.0001), higher NIHSS score (OR = 0.64, 95% CI = 0.61–0.74, *p* = 0.04), larger volume of intracerebral hematoma (OR = 1.01, CI = 1.01–1.02, *p* = 0.01) and higher fibrinogen to albumin ratio (OR = 1.16, CI = 1.02–1.31, *p* = 0.03) upon admission were identified as independent predictors of intrahospital mortality (Table 3). In addition, a fibrinogen to albumin ratio cut-off level of >0.075 upon admission was significantly associated with increased intrahospital mortality in a ROC analysis (Youden’s index = 0.26, sensitivity = 0.51, specificity = 0.77). Furthermore, Spearman correlation indicated a significant correlation between higher fibrinogen to albumin ratio and advanced age (r = 0.24, *p* = 0.0008) as well as higher fibrinogen to albumin ratio and higher NIHSS score (r = 0.38, *p* < 0.0001) upon admission, as shown in Table 4.

## 4. Discussion

### 4.1. Summary of Findings

The aim of the present study was to assess the impact of the fibrinogen to albumin ratio concerning intrahospital mortality on neurosurgical ICU patients with ICH. According to previous studies, advanced age, lower GCS score, higher APACHE II score, higher NIHSS score, and larger volume of intracerebral hematoma upon admission are independent predictors of intrahospital mortality [1,2,4,6,7,8,9,10,11,12,13,34]. Furthermore, the present study identified a higher fibrinogen to albumin ratio as a novel independent and additional predictor of intrahospital mortality in neurosurgical ICU patients with ICH. The fibrinogen to albumin ratio was commonly identified as a predictor of poor outcome and adverse events in patients with cardiovascular diseases, cancer, sepsis, and stroke, although not in neurosurgical ICU-admitted ICH patients [17,18,19,20,21,22,23,24,25,26]. This is the first report on the impact of the fibrinogen to albumin ratio in neurosurgical ICU patients with ICH, and it presents a significant association of intrahospital mortality and a fibrinogen to albumin ratio >0.075 upon admission. This finding could be helpful to improve prognostication of intrahospital mortality and determine whether to initiate or withdraw ICU treatment in neurosurgical ICU-admitted ICH patients.

### 4.2. Intra-Hospital Mortality

The overall intrahospital mortality in the present study is comparable to previous studies, with a rate of 35.4% [1,2,4,6,7,8]. In addition, intrahospital mortality was significantly related with advanced age, male gender, lower initial GCS score, higher APACHE II score, pre-existing medication, consumption of antihypertensive medication, lower body temperature, lower cholinesterase level, lower albumin level, lower level of prothrombin time, and lower rate of infratentorial localization of ICH, and higher rate of conservative medical treatment, larger ICH volume, higher incidence of IVH, higher level of partial thromboplastin time, and higher level of CRP to albumin ratio upon admission, which is consistent with various studies [3,5,6,7,8,9,10,11,12,13,14,16,27,34,35]. Except for body temperature upon admission, no significant difference between the groups in regard to CP within the first 24 h was observed, which emphasized that the same ICU treatment regime was performed in all patients. Moreover, the group of survivors received an additional surgical treatment significantly more frequently, possibly due to an intracranial pressure lowering and life-saving effect of surgery, regardless of neurological outcome. However, this fact could also be a bias due to an early withdrawal of therapy in regard to additional surgical treatment. On account of the retrospective character of the study design, it was not possible to distinguish between the two explanations.

### 4.3. Fibrinogen to Albumin Ratio

The acute-phase protein fibrinogen is an important part of the coagulation system as well as an appropriate marker of systemic inflammation [20,26,27,36]. On this account, fibrinogen level was analyzed as a prognostic marker in patients with according hemorrhagic transformation of acute ischemic stroke and outcome in patients with subarachnoid hemorrhage and ICH [20,26,27,36]. In contrast, the negative acute-phase protein albumin has been identified in several studies as a suitable biomarker in the prediction of inflammatory diseases and mortality after ICH [6,37,38]. Hypoalbuminemia, frequently as an expression of malnutrition and hepatic insufficiency, has been associated with increased intrahospital mortality after community-acquired bloodstream infections with severe sepsis and septic shock as well as ICH [6,37]. In the current study, lower albumin level was the primary factor in the fibrinogen to albumin ratio. In contrast to fibrinogen, hypoalbuminemia could be a result of an acute metabolic stress response after ICH [39,40]. Due to the measurement in the acute phase after ICH upon admission, the lower level of albumin could be an expression of an acute drop in serum albumin level, which may be proportionally to the severity of the ICH [39,40,41,42].

However, both biomarkers, fibrinogen and albumin, are synthesized in the liver, so the prognostic value of these single biomarkers alone is limited in patients with hepatic insufficiency (e.g., cancer, malnutrition, or chronic heart failure). On this account, a new biomarker, the fibrinogen to albumin ratio, was implemented to determine medical conditions including malnutrition, coagulation system, systemic inflammation, and hepatic insufficiency [20,21,22,23,24,25,26].

Nevertheless, the amount and predictive value of the fibrinogen to albumin ratio seems to be dependent on the underlying diseases, as shown by very heterogenous study results [18,19,20,21,22,23,24,25,26]. Several studies have indicated a correlation of elevated fibrinogen to albumin ratio and poor outcome and the presence of adverse events in patients with cardiovascular diseases, cancer, venoarterial extracorporeal membrane oxygenation, and stroke [18,19,20,21,22,23,24,25,26]. In contrast, a lower fibrinogen to albumin ratio has predicted 28-day mortality in peritonitis-induced septic patients and is associated with poor overall survival in esophageal small-cell carcinoma patients [18,19]. Furthermore, heterogenous cut-off levels concerning outcome, presence of adverse events, overall survival, and mortality have been described [18,19,20,24]. Tai et al. proposed that a cut-off level of ≤8.85 for the fibrinogen to albumin ratio predicted a lower overall survival in patients with peritonitis-induced sepsis [19]. In contrast, Xu et al. revealed that the optimal preoperative cut-off level for the fibrinogen to albumin ratio concerning unfavorable overall survival in gallbladder cancer patients was 0.08, while Ruan et al. recommended a cut-off level of ≥9.51 as a predictor for hemorrhagic transformation in patients with acute ischemic stroke [20,24]. This discrepancy can be justified by the heterogeneity in underlying disease and study populations. Patients admitted to the ICU due to infection (e.g., pneumonia or sepsis) or after surgery (e.g., oncological surgery) frequently have higher inflammatory markers and lower albumin level upon admission and, hence, a higher fibrinogen to albumin ratio than neurosurgical ICU patients with ICH, in which loss of consciousness is the main indication for ICU admission.

However, our results indicate that the fibrinogen to albumin ratio upon admission seems to be an appropriate early serum biomarker to predict intrahospital mortality in neurosurgical ICU patients with ICH. The benefit of determination of serum biomarkers, e.g., the fibrinogen to albumin ratio, upon admission, is that they are not affected by medical interventions and express the native health medical conditions of the ICH patients. Additionally, the serum biomarker fibrinogen and albumin are fast and easily to determine from the routine blood samples upon admission. In comparison to time-consuming scores, e.g., the NIHSS score or the APACHE II score, or calculating the ICH volume, the fibrinogen to albumin ratio is a quick way to assess and economical clinical parameter. Nevertheless, the fibrinogen to albumin ratio should not be used as a single predictor of intrahospital mortality. However, in combination with well-known predictors of intrahospital mortality, the fibrinogen to albumin ratio seems to be a new additional parameter not only to improve ICU treatment in terms of initiating an aggressive ICU treatment but also in the early identification of patients with a high risk of intrahospital mortality in future.

### 4.4. Limitations and Strengths of the Study

There are several limitations on the present study, notably its retrospective character, whose problems are well-known; therefore, the findings should be interpreted with caution. Furthermore, due to the retrospective study design, no serial measurement of fibrinogen and albumin, and thus the fibrinogen to albumin ratio, was available. For this reason, there was no further analysis, e.g., the impact of surgical interventions on the fibrinogen to albumin ratio or the influence of a transfusion-associated improved fibrinogen to albumin ratio on the clinical outcome of the patients. The fibrinogen and albumin level may be influenced by many factors of anticoagulation and inflammation, e.g., a surgical intervention. Furthermore, the fibrinogen to albumin ratio could acutely be improved by transfusion of fibrinogen and/or albumin, which are both important drugs in neurosurgical intensive care treatment. The application of fibrinogen might be helpful to reduce the risk of re-bleeding, and albumin is frequently used to increase the oncotic pressure in blood to optimize systolic blood pressure and avoidance of cerebral and/or pulmonary edema. Therefore, the improvement of the fibrinogen to albumin ratio could affect the outcome in a direct manner. However, the aim of the current study was to identify an early serum biomarker, which is easy to determine for risk stratification of intrahospital mortality. Nevertheless, the influence of an improved fibrinogen to albumin ratio on the outcome and mortality of ICH patients as well as the influence of surgical interventions on the fibrinogen to albumin ratio are interesting fields, which should be investigated in a further prospective study. Moreover, this study identified a significant correlation of an initial fibrinogen to albumin ratio of >0.075 and increased intrahospital mortality. However, the influence of the fibrinogen to albumin ratio on the long-term functional outcome of these patients remains still unclear and should investigated in further study. Finally, as patients with ICH due to vascular malformations, malignancy, or trauma were excluded, these results cannot be applied to all kinds of ICH.

The strength of the present study lies in the use of a large study population with comprehensive demographic characteristics as well as clinical, radiological, and laboratory chemistry data records. In addition, to the authors’ knowledge, this is the first study to investigate the impact of the fibrinogen to albumin ratio on intrahospital mortality of neurosurgical ICU patients with ICH.

## 5. Conclusions

In the current study, the fibrinogen to albumin ratio is an independent predictor of intrahospital mortality in neurosurgical ICU patients with ICH. An increased risk of intrahospital mortality was identified in ICH patients with a fibrinogen to albumin ratio greater than 0.075 upon admission. This ratio could be a helpful and appropriate serum biomarker to assist early decision making in regard to initiating or declining further ICU treatment.

## Figures and Tables

**Table 1:** Baseline data, comorbidities and premedication of the study population.

Parameter	Overall (*n* = 198)	Survivor (*n* = 128)	Non-Survivor (*n* = 70)	*p*-Value
**Baseline Data**				
Age, years, mean (±SD) *	68.7 (13)	66.1 (13.1)	73.5 (11.4)	<0.0001
Women, *n* (%) *	87 (43.9)	63 (49.2)	24 (34.3)	0.04
Men, *n* (%) *	111 (56.1)	65 (50.8)	46 (65.7)	
Body Mass Index, kg/m^2^, median (IQR) *	26.2 (24.5–29.4)	27.3 (24.6–29.4)	26 (24.5–27.8)	0.06
Glasgow Coma Scale score, median (IQR) *	6.5 (3–11)	8 (3–13)	4 (3–6)	<0.0001
APACHE II score, median (IQR) *	18 (14–21)	14 (13–17)	20 (18–22)	<0.0001
NIHSS score, median (IQR) *	15 (9–25)	17 (8–19)	26 (18–31)	<0.0001
mRS score, median (IQR) *	5 (3–5)	4 (3–5)	5 (4–5)	0.06
Hospital stay, median (IQR) **	16.5 (5–31)	24 (16–38)	3 (1–9)	<0.0001
**Comorbidities**				
Chronic arterial hypertension, *n* (%) *	68.7 (13)	82 (64.1)	37 (52.9)	0.12
Chronic obstructive pulmonary diseases, *n* (%) *	87 (43.9)	19 (14.8)	5 (7.1)	0.11
Cardiac arrhythmia, *n* (%) *	111 (56.1)	26 (20.3)	17 (24.3)	0.52
Coronary artery disease, *n* (%) *	26.2 (24.5–29.4)	16 (12.5)	14 (20)	0.16
Heart failure, *n* (%) *	6.5 (3–11)	6 (4.7)	7 (10)	0.15
History of cardiac/cardiosurgical intervention, *n* (%) *	18 (14–21)	14 (10.9)	10 (14.3)	0.49
Chronic renal insufficiency, *n* (%) *	15 (9–25)	12 (9.4)	5 (7.1)	0.59
Diabetes mellitus, *n* (%) *	5 (3–5)	19 (14.8)	13 (18.6)	0.5
History of ischemic stroke, *n* (%) *	16.5 (5–31)	17 (13.3)	8 (11.4)	0.71
History of ICH, *n* (%) *	68.7 (13)	4 (3.1)	4 (5.7)	0.38
History of cancer, *n* (%) *	87 (43.9)	11 (8.6)	8 (11.4)	0.52
**Premedication**				
Pre-existing medication, *n* (%) *	102 (51.1)	75 (58.6)	27 (38.6)	0.007
Antihypertensive drugs, *n* (%) *	84 (42.4)	62 (48.4)	22 (31.4)	0.02
Antiobstructive drugs, *n* (%) *	4 (2)	1 (0.8)	3 (4.3)	0.09
Antidiabetic drugs, *n* (%) *	18 (9.1)	8 (6.3)	10 (14.3)	0.06
Antiplatelet agents, *n* (%) *	30 (15.2)	17 (13.3)	13 (18.6)	0.32
New oral anticoagulants, *n* (%) *	11 (5.6)	6 (4.7)	5 (7.1)	0.47
Vitamin K antagonist, *n* (%) *	42 (21.2)	26 (20.3)	16 (22.9)	0.68

SD: standard deviation, IQR: interquartile range, APACHE II: Acute Physiology and Chronic Health Evaluation II, NIHSS: The National Institutes of Health Stroke Scale. * upon admission, ** within the first 24 h.

**Table 2:** Clinical, radiological and treatment parameter of the study population.

Parameter	Overall (*n* = 198)	Survivor (*n* = 128)	Non-Survivor (*n* = 70)	*p*-Value
**Cardiopulmonary parameter**				
Norepinephrine application rate, µg/kg/min, mean (±SD) **	0.03 (0.05)	0.03 (0.04)	0.03 (0.05)	0.47
Systolic blood pressure, mmHg, median (IQR) **	136 (127–143)	135 (128–142)	136 (126–145)	0.27
Heart rate, beats per minute, median (IQR) **	75 (63–88)	75 (64.3–88)	75.5 (60–87.3)	0.33
Inspiratory oxygen fraction, mean (±SD) **	37.5 (13.5)	37.3 (14.1)	37.9 (12.3)	0.77
Intubated patients, *n* (%) *	141 (71.2)	87 (68)	54 (77.1)	0.17
PEEP level, median (IQR) **	8 (6–9)	7 (6–9)	8 (6–10.5)	0.42
Arterial oxygen partial pressure (mmHg), median (IQR) **	109 (99–125)	108.5 (98.3–123.8)	109 (99–125.3)	0.82
Body temperature, centigrade, median (IQR) *	36.2 (35.3–36.8)	36.3 (35.7–36.8)	35.8 (34.8–36.5)	<0.0001
**Serum Biomarker**				
White blood cells, giga/L, mean (±SD) *	11.3 (4.8)	11.1 (4.4)	11.5 (5.4)	0.56
Hemoglobin, g/dL, mean (±SD) *	12.9 (2.1)	13 (2)	12.9 (2.2)	0.6
Hematocrit, %, mean (±SD) *	0.38 (0.06)	0.38 (0.05)	0.38 (0.06)	0.9
Cholinesterase, U/L, mean (±SD) *	7750.8 (2423.3)	8087.7 (2389.3)	7134.79 (2379.8)	0.008
Blood glucose, mg/dL, mean (±SD) *	168 (66.3)	161.7 (62)	178.7 (72.8)	0.09
Serum lactate, mmol/L, mean (±SD) *	1.9 (1.8)	1.8 (1.7)	2.1 (1.9)	0.37
Troponin I, µg/dL, mean (±SD) *	0.35 (2.9)	0.76 (0.4)	0.71 (0.5)	0.53
Cortisol, µg/dL, mean (±SD) *	28.1 (21.2)	0.37 (0.5)	0.43 (0.5)	0.44
C-reactive protein, mg/L, mean (±SD) *	25.2 (44)	21 (42.4)	32.8 (46)	0.07
Albumin, g/L, mean (±SD) *	38 (5.8)	38.9 (5.1)	36.3 (6.7)	0.002
C-reactive protein to albumin ratio, mean (±SD) *	0.7 (1.3)	0.6 (1.2)	1 (1.5)	0.02
Thyroid stimulating hormone, mU/L, mean (±SD) *	1.3 (1.1)	1.4 (1.1)	1.2 (0.7)	0.56
Prothrombin time, %, mean (±SD) *	82.7 (26.7)	86.8 (25)	75.1 (28.3)	0.003
Partial thromboplastin time, seconds, mean (±SD) *	32.8 (12.7)	31.1 (9.5)	36 (16.7)	0.01
Antithrombin III, %/NORM, mean (±SD) *	89.2 (15.4)	90.1 (14.6)	87.5 (16.8)	0.26
Fibrinogen, g/L, mean (±SD) *	3.3 (1.1)	3.2 (1.1)	3.5 (1.1)	0.1
Fibrinogen to albumin ratio, mean (±SD) *	0.9 (0.03)	0.08 (0.3)	0.1 (0.04)	0.002
**Treatment**				
Medical treatment, *n* (%)	67 (33.8)	34 (26.6)	33 (47.1)	0.003
Additional Surgical Treatment, *n* (%)	131 (66.2)	94 (73.4)	37 (52.9)	
Insertion EVD, *n* (%)	41 (31.2)	28 (29.8)	13 (35.1)	0.55
Evacuation ICH, *n* (%)	45 (34.4)	32 (34)	13 (35.1)	0.91
Decompressive craniectomy, *n* (%)	14 (10.7)	9 (9.6)	5 (13.5)	0.51
Decompressive craniectomy and evacuation ICH, *n* (%)	31 (23.7)	25 (26.6)	6 (16.2)	0.21
**Computed tomography scan**				
Localization				
Supratentorial, lobar, *n* (%) *	67 (33.8)	42 (32.8)	25 (35.7)	0.68
Supratentorial, deep, *n* (%) *	96 (48.5)	58 (45.3)	38 (54.3)	0.23
Infratentorial, *n* (%) *	35 (17.7)	28 (21.9)	7 (10)	0.04
ICH volume, cm^3^, mean (±SD)	61.2 (46.6)	51.8 (38.3)	78.5 (55.2)	<0.0001
IVH, *n* (%) *	142 (71.7)	85 (66.4)	57 (81.4)	0.03
Hydrocephalus, *n* (%) *	102 (51.5)	59 (46.1)	43 (61.4)	0.14

SD: standard deviation, IQR: interquartile range, PEEP: positive end expiratory pressure, EVD: external ventricular drain, ICH: intracerebral hemorrhage, IVH: intraventricular hemorrhage. * upon admission, ** within the first 24 h.

**Table 3:** Independent predictors of intrahospital mortality after spontaneous ICH.

Parameter	Odds Ratio	95% CI	*p*-Value
Age, years, mean (±SD) *	1.07	1.03–1.1	<0.0001
Glasgow Coma Scale score, median (IQR) *	0.75	0.76–0.85	<0.0001
APACHE II score, median (IQR) *	0.71	0.63–0.81	<0.0001
NIHSS score, median (IQR) *	0.64	0.61–0.74	0.04
Sex, (*n*) *	0.77	0.3–1.9	0.58
Pre-existing medication, *n* (%) *	2.46	0.91–6.66	0.08
Body temperature, centigrade, median (IQR) *	0.85	0.58–1.24	0.4
Cholinesterase, U/L, mean (±SD) *	1.0	0.99–1	0.2
Albumin, g/L, mean (±SD) *	1.02	0.92–1.14	0.66
Prothrombin time, %, mean (±SD) *	0.99	0.97–1.02	0.83
Partial thromboplastin time, %/NORM, mean (±SD) *	1.02	0.92–1.08	0.66
C-reactive protein to albumin ratio, mean (±SD) *	0.74	0.47–1.15	0.18
Fibrinogen to albumin ratio, mean (±SD) *	1.16	1.02–1.31	0.03
ICH volume, cm^3^, mean (±SD) *	1.01	1.01–1.02	0.01
IVH, *n* (%) *	0.89	0.29–2.68	0.83

95% CI: 95% confidence interval, SD: standard deviation, IQR: interquartile range, APACHE II: Acute Physiology and Chronic Health Evaluation II, NIHSS: The National Institutes of Health Stroke Scale, ICH: intracerebral hemorrhage, IVH: intraventricular hemorrhage. * upon admission.

**Table 4:** Correlation of fibrinogen to albumin ratio with all independent predictors of intrahospital mortality.

Parameter	Correlation Coefficient r	*p*-Value
Fibrinogen to albumin ratio/Age *	0.24	0.0008
Fibrinogen to albumin ratio/GCS *	0.002	0.97
Fibrinogen to albumin ratio/APACHE II score *	−0.12	0.08
Fibrinogen to albumin ratio/NIHSS score *	0.38	<0.0001
Fibrinogen to albumin ratio/Volume of intracerebral hemorrhage *	0.05	0.51

APACHE II: Acute Physiology and Chronic Health Evaluation II, NIHSS: The National Institutes of Health Stroke Scale. * upon admission.

## Data Availability

The data presented in this study are available on request from the corresponding author.
